# Supplementary figures and images for: Daily Variability in Sedentary Behaviour and Physical Activity Responsiveness in Older Women
Source: Sensors (Basel). 2025 Mar 30;25(7):2194. doi: 10.3390/s25072194 (PMC11991520; doi:10.3390/s25072194)

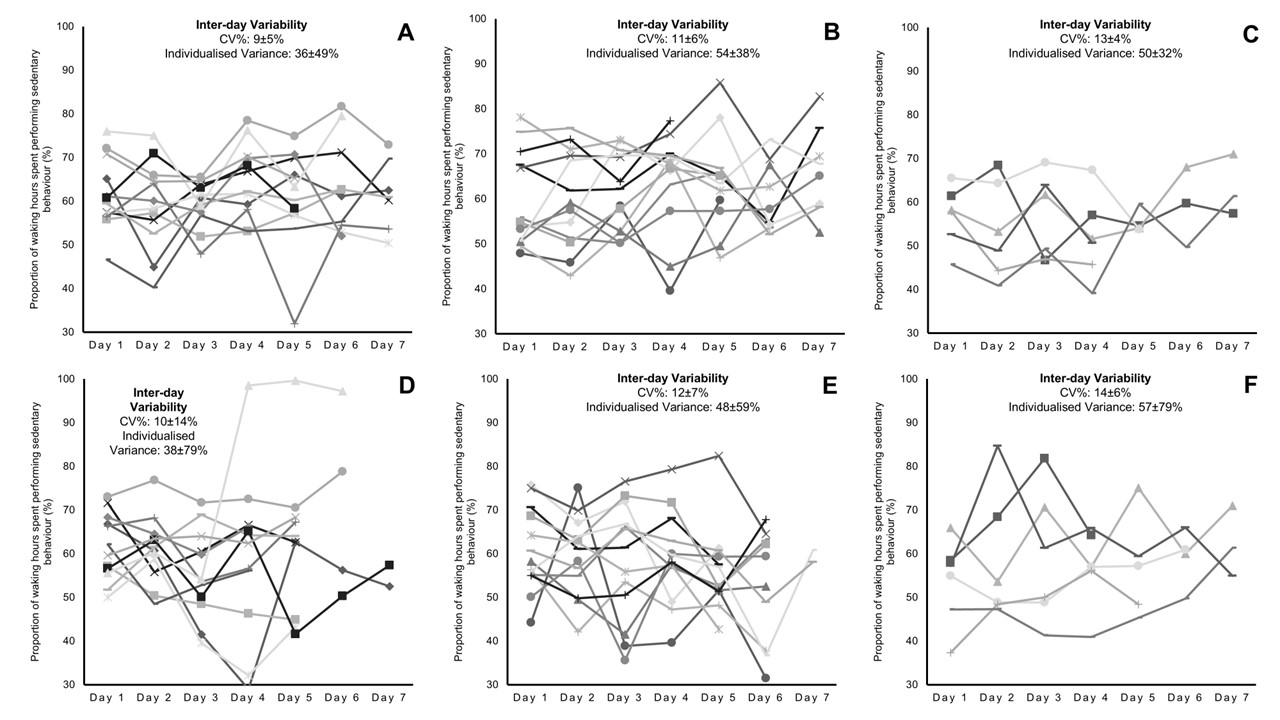

Supplement: Supplementary file 1 [file sensors-25-02194-s001.zip › Grant et al Appendix Figure 2R3.jpg]

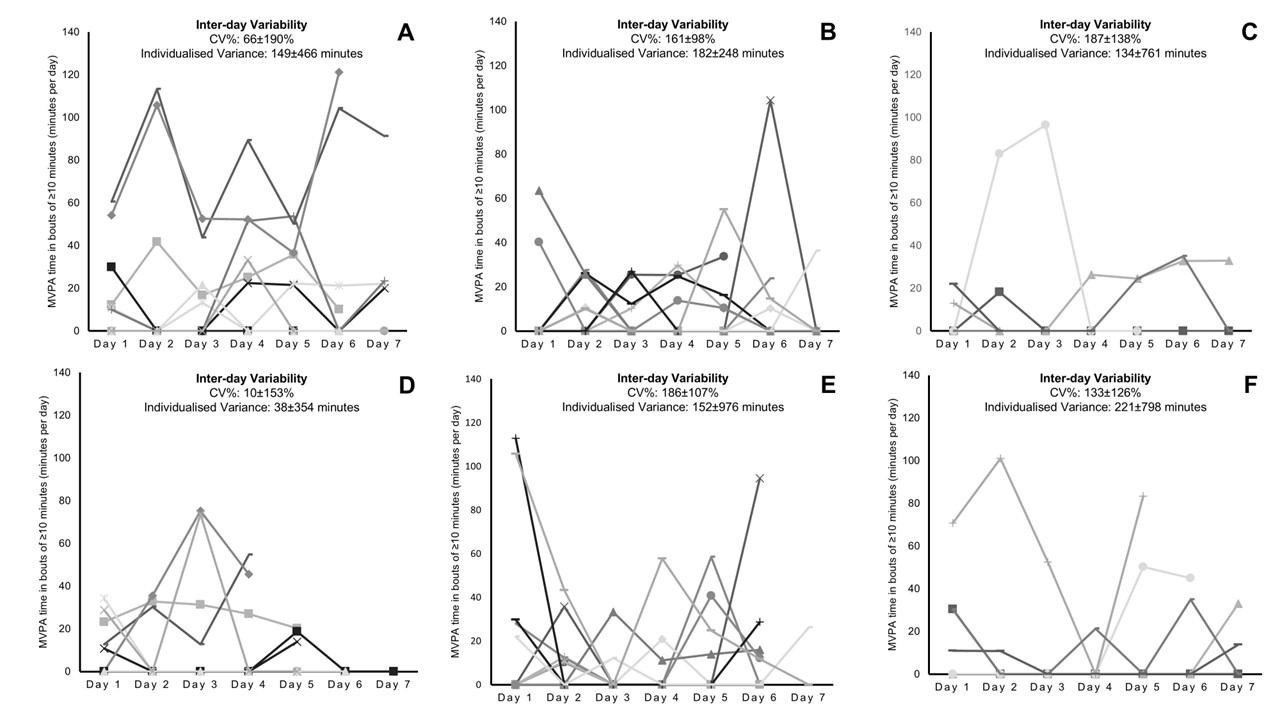

Supplement: Supplementary file 1 [file sensors-25-02194-s001.zip › Grant et al Appendix Figure 1R3.jpg]
